# Prediction of Postpartum Haemorrhage After Labour Induction: An Internally Validated 10-Hour Risk-Stratification Threshold

**DOI:** 10.3390/diagnostics16121910

**Published:** 2026-06-19

**Authors:** Sait Erbey, Ömer Osman Eroğlu, Mehmet Alican Sapmaz, Bilge Erbey, Murat Polat, Cansın Eroğlu, Çağanay Soysal

**Affiliations:** 1 Department of Obstetrics and Gynecology, Ankara Etlik City Hospital, 06170 Ankara, Turkey; 2 Independent Obstetrics and Gynecology Specialist, 06170 Ankara, Turkey

**Keywords:** postpartum haemorrhage, labour induction, induction duration, clinical prediction model, decision curve analysis, nomogram

## Abstract

**Background/Objectives:** Postpartum haemorrhage (PPH) remains a leading cause of maternal morbidity, and the relationship between labour-induction duration and haemorrhagic risk has not been translated into a practical intrapartum risk-stratification framework. We aimed to derive a clinically interpretable induction-duration threshold for PPH risk stratification and to explore an internally validated parsimonious clinical decision-support model. **Methods:** In this retrospective cohort of 1128 induced singleton labours at ≥37 weeks at a Turkish tertiary centre, laboratory-defined PPH was operationalised as a haemoglobin drop ≥ 2 g/dL. Multivariable logistic regression identified independent predictors; receiver operating characteristic (ROC) analysis with 2000-replicate bootstrap internal validation derived a duration threshold; calibration, decision curve analysis, and a probability-scaled nomogram were additionally evaluated. **Results:** PPH occurred in 143 patients (12.7%). Four predictors were independently associated with PPH: induction duration (adjusted odds ratio 1.243 per hour; 95% CI 1.191–1.298; *p* < 0.001), parity, emergency caesarean, and maternal age. Induction duration achieved an apparent area under the curve of 0.773 (optimism-corrected 0.773); a Youden-optimal threshold of 10.1 h yielded 86.7% (95% CI 80.2–91.3%) sensitivity and 96.8% (95% CI 95.0–97.9%) negative predictive value. The model showed favourable calibration and positive net clinical benefit on decision curve analysis. **Conclusions:** Induction duration was independently associated with PPH; the internally validated 10.1 h threshold therefore represents a hypothesis-generating risk-stratification benchmark—reflecting an association rather than a demonstrated causal effect—that requires prospective external validation in independent populations before clinical application. Whether this association is causal or reflects an underlying myometrial phenotype requires prospective study.

## 1. Introduction

Postpartum haemorrhage (PPH) remains a leading cause of preventable maternal morbidity and mortality despite universal adoption of active third-stage management and prophylactic uterotonics. Obstetric haemorrhage—of which postpartum haemorrhage represents the largest fraction—accounts for approximately 27% of direct maternal mortality worldwide [1] and continues to affect 1–5% of deliveries even in high-income settings with robust obstetric infrastructure [2]. The persistence of PPH as a leading cause of maternal death despite protocol-level interventions indicates that individual patient- and labour-level risk factors remain incompletely characterised and insufficiently acted upon at the bedside [3]. In Turkey, a population-based national study reported 779 maternal deaths between 2012 and 2015 (Maternal Mortality Ratio 19.7 per 100,000 live births), with obstetric haemorrhage identified as a leading preventable direct cause, underscoring that this is not solely a low-resource problem [4].

Over the past two decades, induction of labour (IOL) has become one of the most frequently performed obstetric interventions. Current estimates place induction rates at 25–35% in high-income countries [5], further reinforced by randomised evidence supporting elective 39-week induction in low-risk nulliparous women [6], and substantially higher proportions in upper-middle-income countries [7]. The clinical impetus for this trend is well founded: indications such as late-term pregnancy, premature rupture of membranes, and gestational diabetes each carry independent foetal and maternal risks that induction is intended to mitigate. Yet the intervention itself introduces a distinct haemorrhagic exposure. Prolonged exogenous oxytocin exposure has been associated with impaired third-stage uterine contractility and uterine atony [8,9], a risk that may be compounded by the dysfunctional labour patterns and higher operative-delivery rates that often accompany prolonged induction. Mechanical cervical ripening devices—particularly the intracervical Foley balloon catheter—add a distinct biological dimension: transcervical balloon insertion triggers local prostaglandin E2 release and pro-inflammatory cytokine upregulation that, while effective for cervical maturation, may independently perturb myometrial contractile homeostasis and predispose to third-stage haemorrhage [10,11].

The relationship between induction and PPH has been examined in several observational studies and meta-analyses, yet important questions remain unanswered. First, the contribution of individual induction agents—particularly mechanical devices such as the Foley balloon catheter versus pharmacological agents—has not been consistently delineated after adjustment for case-mix confounders [11,12]. Second, while prolonged induction is biologically plausible as a dose–response risk factor, no practical, ROC-validated duration threshold has been established that can stratify postpartum monitoring intensity in real time [13,14].

We therefore designed the present study with three a priori objectives: (1) to determine the incidence and severity distribution of PPH in a cohort of induced labours at a single high-volume Turkish centre; (2) to identify independent predictors of PPH using multivariable logistic regression and to validate these findings across pre-specified sensitivity and subgroup analyses stratified by parity, BMI, delivery mode, oxytocin-only exposure, and baseline anaemia; and (3) to derive and internally validate the discriminative performance of a clinically interpretable induction-duration threshold. We anticipated that total induction duration—as a surrogate for cumulative uterine stimulation exposure—would be associated with an increased risk of PPH. Beyond threshold derivation, we additionally developed and internally validated a parsimonious four-predictor clinical decision-support model—comprising routinely available intrapartum variables—as an exploratory tool to facilitate individualised risk stratification, with full acknowledgement that external validation is required before clinical adoption.

## 2. Materials and Methods

### 2.1. Study Design and Setting

This retrospective cohort study was conducted at the Department of Obstetrics and Gynecology, Ankara Etlik City Hospital—a 4000-bed public tertiary referral centre performing 13,089 total deliveries in 2025, of which 8431 occurred in the main delivery suite during the study period, and serving as the primary obstetric facility for the Ankara metropolitan area. All induction cases between 1 January and 31 December 2025 were screened for eligibility. The study was approved by the Clinical Research Ethics Committee of Ankara Etlik City Hospital (approval reference: AEŞH-BADEK2-2026-346; approval date: 21 April 2026) in conformity with the Declaration of Helsinki. Because the dataset comprised routinely collected, anonymized clinical records accessible to the investigators within their institutional clinical practice, formal ethics committee approval was sought specifically for academic publication and secondary research use prior to manuscript preparation, in accordance with institutional and national guidance on retrospective use of clinical data. The requirement for individual informed consent was waived by the ethics committee given the retrospective, anonymised nature of the data. Patient identifiers were replaced with anonymous numeric codes prior to analysis. Reporting conforms to the Strengthening the Reporting of Observational Studies in Epidemiology (STROBE) checklist for cohort studies (Supplementary Material S1) [15].

### 2.2. Eligibility Criteria and Institutional Induction Protocol

Women were eligible for inclusion if they underwent induction of labour with any pharmacological or mechanical agent—oxytocin intravenous infusion, dinoprostone vaginal insert (Propess 10 mg), intracervical Foley balloon catheter (16 Fr, 30 mL inflated with sterile saline, removed after 12 h or with spontaneous expulsion), or a combination thereof—and had a singleton, live pregnancy at ≥37 + 0 weeks of gestation. Per institutional protocol, elective induction for late-term pregnancy was scheduled at 41 + 0 weeks; induction for oligohydramnios was triggered at a deepest vertical pocket < 2 cm; induction for abnormal biophysical profile required BPP ≤ 6/10 on two occasions. The single-balloon 16 Fr Foley catheter represents the standard mechanical ripening agent in our institution; double-balloon catheters were not routinely available during the study period. Oxytocin was initiated at 2 mU/min and titrated every 30 min to adequate uterine activity (maximum 20 mU/min), following the institutional low-dose protocol. Exclusion criteria applied to induction candidates admitted to the main delivery suite were: (i) previous caesarean delivery (at our institution, all patients with a prior caesarean are managed with planned repeat caesarean rather than trial of labour, as TOLAC is not part of routine practice, and such patients are not screened for induction); (ii) pre-gestational diabetes or insulin-requiring gestational diabetes, which are managed under individualised, non-standard induction pathways at our institution; (iii) coagulopathy or thrombocytopenia (platelet count < 100 × 10^9^/L); (iv) preterm induction (<37 + 0 weeks); and (v) incomplete medical records precluding reliable extraction of induction duration or peri-delivery haemoglobin values. Patients managed under the institutional perinatology service—including those with severe pre-eclampsia or HELLP syndrome, placenta praevia or accreta spectrum disorder, multiple gestation, foetal growth restriction, and major congenital anomalies—are cared for in a separate high-risk unit and were therefore not part of the main-delivery-suite induction population screened for this study.

### 2.3. Outcomes

The primary outcome was laboratory-defined PPH, operationalised as a haemoglobin (Hb) drop of ≥2 g/dL between the pre-delivery measurement and the first routine postpartum sample obtained within 6–24 h after delivery according to institutional protocol. Although current American College of Obstetricians and Gynecologists (ACOG) and International Federation of Gynecology and Obstetrics (FIGO) definitions preferentially use cumulative blood loss ≥ 1000 mL or signs of hypovolaemia [9,14], these volumetric criteria rely on prospective quantitative blood loss measurement that was not uniformly implemented during the study period and could not be reliably reconstructed from the retrospective record. We therefore adopted the Hb drop ≥ 2 g/dL criterion, which has direct empirical support as a marker concordant with postpartum haemorrhage diagnosis [16], correlates well with clinically meaningful haemorrhage, and avoids the well-documented underestimation and inaccuracy of visual blood loss estimation, particularly at higher blood-loss volumes [17]. A pre-specified sensitivity outcome—severe PPH—was defined as an Hb drop of ≥3 g/dL. All women received routine prophylactic oxytocin (10 IU intramuscular or slow intravenous) as part of active third-stage management. Secondary outcomes included blood transfusion (any packed red blood cell unit), additional uterotonic administration beyond prophylactic oxytocin, and intensive care unit (ICU) transfer. Crucially, additional uterotonic administration was recorded as a secondary outcome only and was not part of the primary PPH definition; additional uterotonics were administered at the clinician’s discretion in response to observed postpartum bleeding. For clarity, total induction duration was defined as the time from placement or initiation of the first induction agent—mechanical (Foley balloon) or pharmacological (dinoprostone insert or oxytocin infusion), whichever was earliest—to the time of delivery, recorded in minutes and converted to hours; it therefore encompasses the entire induction process rather than the active phase alone.

### 2.4. Data Collection

Data were extracted from electronic hospital records and supplementary paper charts by two obstetrics and gynaecology specialists (S.E., M.A.S.) working independently; discrepancies were resolved by a third reviewer (Ç.S.). Variables were collected across five domains: (1) maternal and obstetric characteristics (age, parity, gravidity, BMI, prior caesarean count); (2) induction-related parameters (indication, gestational age at induction, initial Bishop score, agent[s], maximum oxytocin dose in mU/min, and total induction duration—defined as the interval in hours from administration of the first induction agent [oxytocin infusion start, Propess insertion, or Foley balloon placement, whichever was earliest] to delivery of the infant; this composite interval deliberately encompasses all phases of the induction process to reflect total uterine stimulation exposure, while acknowledging that cumulative oxytocin dose—rather than total duration alone—represents the most proximate mechanistic correlate of oxytocin-receptor desensitisation and was not available for this retrospective cohort); (3) labour and delivery data (active labour duration from 6 cm dilatation to full dilatation, second-stage duration, mode of delivery, episiotomy, perineal laceration grade, and mode of placental separation); (4) haemorrhagic outcomes (pre-delivery and postpartum Hb concentrations, transfusion units, additional uterotonic agent[s], and ICU transfer); and (5) neonatal outcomes (birth weight, Apgar scores at 1 and 5 min). Umbilical cord gas and NICU admission data were not uniformly captured and are acknowledged as a limitation.

### 2.5. Statistical Analysis

Because the cohort size was fixed by the calendar-year inclusion window (1 January –31 December 2025), adequacy of sample size for multivariable modelling was verified post hoc against two complementary criteria: (a) the classical events-per-variable (EPV) ≥ 10 rule applied to binary logistic regression with a reported PPH incidence of 10–15% in comparable obstetric cohorts [18,19], and (b) the shrinkage-based sample-size criteria proposed by Riley and colleagues [20]. The final cohort of 1128 cases with 143 PPH events yielded EPV = 14.3 for the full 10-predictor model and EPV = 35.8 for the parsimonious 4-predictor model, exceeding both thresholds. For the severe-PPH sensitivity analysis (43 events), EPV declined to approximately 10.75 in the parsimonious model—at the lower bound of conventionally acceptable power; results from this sub-analysis are accordingly interpreted with appropriate caution. Because cases with incomplete records were excluded at eligibility screening, no missing data remained in the final analytical cohort (Supplementary Figure S1, STROBE flow diagram).

Continuous variables were assessed for normality using visual inspection of histograms and Q-Q plots together with skewness and kurtosis examination; the Shapiro–Wilk test was interpreted cautiously because, in the present sample size, it is known to flag trivial deviations from normality. Variables with approximately normal distributions (age: skewness 0.18, kurtosis −0.23; pre-delivery Hb: skewness 0.03, kurtosis −0.19; total induction duration: skewness 0.47, kurtosis −0.20; initial Bishop score: skewness 0.20, kurtosis −0.52; gestational age at induction) are reported as mean ± SD and compared with Student’s *t*-test; all others as median (Q1–Q3) and compared with the Mann–Whitney U test. Categorical variables were analysed with the chi-square or Fisher’s exact test. Because our univariable screening was pre-specified and hypothesis-driven rather than hypothesis-generating, formal multiple-comparison adjustment was not applied; *p*-values should be interpreted cautiously within this predefined analytical framework.

Variables reaching *p* < 0.10 on univariable analysis (age, parity, total induction duration, mode of delivery, induction agent [overall], epidural analgesia, and manual placental delivery) were entered into a multivariable binary logistic regression model (enter method), together with BMI, birth weight, pre-delivery Hb, and Bishop score, which were included as a priori clinical covariates based on their established associations with PPH risk in the literature. Epidural analgesia and manual placental delivery were considered downstream mediators of the haemorrhagic event rather than antecedent risk factors—manual placental removal in particular is performed in response to retained placenta or active bleeding, both of which represent intermediate manifestations of PPH rather than upstream exposures—and were therefore excluded from the principal adjusted model on pre-specified biological grounds. Results are expressed as adjusted ORs with 95% CIs. A parsimonious final model was subsequently derived by retaining only variables that reached statistical significance after full adjustment; the complete regression output (coefficients, standard errors, Wald statistics) is reported in the Results section. The assumption of logit-linearity for continuous predictors (induction duration, age, parity, BMI, birth weight) was assessed by examining standardised residuals against predicted logits and by inspecting odds-ratio patterns across categorical strata; no marked non-linear deviations were detected for the four parsimonious-model predictors, although the non-linear pattern observed for BMI is acknowledged in the Limitations. Model fit was assessed by the Nagelkerke pseudo-R^2^ and Hosmer–Lemeshow goodness-of-fit test, with the caveat that the latter may be oversensitive to trivial miscalibration in large samples; calibration was therefore additionally described by calibration slope, intercept, Brier score, and a decile-based calibration plot. Collinearity was assessed by mean-centred variance inflation factors (VIFs) and tolerance values. A multiplicative interaction between induction duration and Foley balloon use was tested.

The area under the ROC curve (AUC) for induction duration was calculated with 95% CI by bootstrap resampling (2000 replicates); the optimal threshold was identified by maximising the Youden index, and sensitivity, specificity, positive predictive value (PPV), negative predictive value (NPV), and likelihood ratios were derived. Internal validation of both AUC and calibration of the final multivariable model was performed using 2000-replicate bootstrap with Harrell’s optimism-correction procedure: in each bootstrap replicate the model was refitted on the resampled data; performance was evaluated both in the bootstrap sample and back-applied to the original cohort, and the difference (optimism) was averaged across replicates and subtracted from the apparent estimate to yield optimism-corrected metrics for both discrimination (AUC) and calibration (slope, intercept, and Brier score). Threshold stability was examined by summarising the bootstrap distribution of the Youden-optimal cut-off across bootstrap replicates. To quantify the robustness of observed associations to unmeasured confounding, E-values were calculated for the principal predictors (induction duration, emergency caesarean, and parity) using the VanderWeele–Ding framework [21].

Pre-specified sensitivity analyses included: (i) restriction to vaginal deliveries only (*n* = 789); (ii) redefinition of the outcome as severe PPH (Hb drop ≥ 3 g/dL); (iii) restriction to oxytocin-monotherapy exposure (*n* = 571) to isolate the duration–PPH relationship from confounding by mechanical ripening agents; and (iv) a post hoc sensitivity model in which epidural analgesia was added to the parsimonious 4-predictor model, to verify that excluding this potential intermediate variable did not materially affect the duration estimate. Exploratory subgroup analyses were carried out by parity (nulliparous vs. multiparous), BMI category, delivery mode (vaginal vs. emergency caesarean), and baseline anaemia status (pre-delivery Hb < 11 vs. ≥11 g/dL). All tests were two-tailed with significance set at *p* < 0.05. Analyses were performed using IBM SPSS Statistics version 27.0 (IBM Corp., Armonk, NY, USA), including the bootstrap module for internal validation, and MedCalc Statistical Software version 22.0 (MedCalc Software Ltd., Ostend, Belgium) for ROC analysis. E-values were computed using the online calculator developed by VanderWeele and colleagues (https://www.evalue-calculator.com, accessed on 17 June 2026) [21]. In addition, an exploratory tertile-based subgroup analysis stratified the cohort by pre-delivery haemoglobin (lowest, middle, and highest tertiles), with within-tertile ROC analyses, optimal-threshold determination, and adjusted odds ratios derived from the parsimonious 4-predictor model. Heterogeneity of the duration effect across tertiles was formally tested by a likelihood-ratio test for the duration × Hb-tertile interaction term.

To assess the clinical utility of the parsimonious model beyond conventional discrimination and calibration metrics, decision curve analysis (DCA) was performed using the method of Vickers and Elkin [22]. Net benefit was computed across threshold probabilities ranging from 1% to 50% and compared against two reference strategies—treating all patients and treating none—as well as against a single-predictor model based on induction duration alone. Calibration and DCA results are reported in the Results section.

Reporting of the multivariable model and its internal validation followed principles consistent with the TRIPOD framework for clinical prediction-model studies, where applicable to a single-cohort, retrospectively analysed development study without external validation. To translate the parsimonious model into a bedside-applicable clinical tool, a probability-scaled nomogram was constructed using the regression coefficients of the four-predictor model. Because delivery mode is ascertained only at the end of labour, the complete four-predictor probability is intended primarily for immediate post-delivery risk stratification, whereas induction duration alone may serve as a dynamic intrapartum marker during the active induction process. Accordingly, the full model is not intended for early intrapartum prediction prior to delivery-mode ascertainment, and should be interpreted strictly as a post-delivery risk-stratification framework rather than a prospective intrapartum prediction model. The model is fully specified by the linear predictor logit(P[PPH]) = −6.115 + 0.218 × induction duration (hours) + 0.041 × maternal age (years) + 0.232 × parity + 0.423 × emergency caesarean (0 = no, 1 = yes), with the predicted probability obtained as P(PPH) = 1/(1 + exp[−logit]). Complete coefficient estimates with standard errors, Wald statistics, and 95% confidence intervals are reported in the Results section.

## 3. Results

A total of 1128 induction cases met the inclusion criteria (Supplementary Figure S1). Mean maternal age was 28.0 ± 5.0 years, median BMI was 26.8 kg/m^2^ (IQR 23.5–29.7), and 47.0% were nulliparous. Late-term pregnancy was the leading indication (45.2%, *n* = 510), followed by PROM (30.1%, *n* = 340), oligohydramnios (11.5%, *n* = 130), abnormal BPP (10.6%, *n* = 120), and diet/metformin-managed gestational diabetes (2.5%, *n* = 28). Oxytocin monotherapy was used most frequently (50.6%, *n* = 571), followed by Propess (19.9%, *n* = 225), oxytocin plus Foley balloon (11.5%, *n* = 130), Foley balloon alone (9.6%, *n* = 108), and oxytocin plus Propess (8.3%, *n* = 94). Delivery was vaginal in 69.9% of cases (*n* = 789) and by emergency caesarean in 30.1% (*n* = 339). The mean total induction duration was 10.5 ± 4.5 h, and the median birth weight was 3297 g (IQR 3017–3588). Full baseline characteristics stratified by PPH status are presented in Table 1.

PPH occurred in 143 patients (12.7%), and severe PPH (Hb drop ≥ 3 g/dL) in 43 (3.8%). The median Hb drop was 2.6 g/dL (IQR 2.3–3.2) in the PPH group versus 0.9 g/dL (IQR 0.6–1.2) in the non-PPH group (*p* < 0.001). Blood transfusion was required in 21 PPH cases (14.7%), corresponding to 1.9% of the entire cohort, with a median of 2 units transfused (range 1–3). Six patients required ICU transfer (0.5% of the full cohort), all from the PPH group. Comparative obstetric and induction parameters are displayed in Table 2.

Additional uterotonics were administered to 95 patients in the PPH group (66.4%)—methylergometrine monotherapy in 53, misoprostol monotherapy in 16, carbetocin in 18, and methylergometrine–misoprostol combination in eight—compared with 54 patients in the non-PPH group (5.5%; *p* < 0.001). Manual placental delivery was performed in 100 cases (8.9%) and was associated with a significantly higher PPH rate than spontaneous delivery (22.0% vs. 11.8%; *p* = 0.005). Among vaginal deliveries, episiotomy was performed in similar proportions of PPH (65.1%) and non-PPH cases (66.6%; *p* = 0.882), whereas third- or fourth-degree perineal laceration occurred in 52 cases (6.6% of vaginal deliveries) and carried a higher PPH rate than lower-grade injuries (23.1% vs. 10.0%; *p* = 0.007). Neonatal outcomes did not differ significantly between groups: median Apgar scores at 1 and 5 min were 9 in both groups (*p* = 0.400 and *p* = 0.952, respectively).

On univariable analysis, seven variables reached *p* < 0.10: age (OR 1.038 per year; *p* = 0.038), parity (OR 1.161 per unit; *p* = 0.027), total induction duration (OR 1.234 per hour; *p* < 0.001), mode of delivery (emergency caesarean OR 1.652; *p* = 0.007), induction agent (overall χ^2^ = 17.5; *p* = 0.002), epidural analgesia (OR 2.020; *p* = 0.022), and manual placental delivery (OR 2.114; *p* = 0.004). BMI, birth weight, pre-delivery Hb, and Bishop score were additionally entered into the multivariable model as a priori clinical covariates, whereas epidural analgesia and manual placental delivery—considered downstream mediators on pre-specified biological grounds—were excluded. After full adjustment, four variables emerged as independent predictors of PPH: total induction duration (aOR 1.243 per hour; 95% CI 1.191–1.298; *p* < 0.001), parity per unit increment (aOR 1.262; 95% CI 1.090–1.460; *p* = 0.002), emergency caesarean delivery (aOR 1.527; 95% CI 1.031–2.261; *p* = 0.035), and maternal age per year (aOR 1.041; 95% CI 1.003–1.082; *p* = 0.037). Neither BMI (aOR 1.005 per kg/m^2^; *p* = 0.831), birth weight (aOR 1.192 per 500 g; *p* = 0.145), Foley balloon-containing agent (aOR 1.580; *p* = 0.131), Propess-containing agent (aOR 1.411; *p* = 0.253), nor pre-delivery Hb (aOR 1.113; *p* = 0.180) reached statistical significance after full adjustment. Initial Bishop score was borderline (aOR 1.135; 95% CI 1.000–1.289; *p* = 0.050). The parsimonious four-predictor model (duration + age + parity + emergency caesarean) achieved an apparent AUC of 0.789, a Nagelkerke R^2^ of 0.205, and a Brier score of 0.098. All mean-centred VIFs were <1.01 (tolerance > 0.99), indicating no meaningful collinearity. Univariable and multivariable logistic regression results are summarised in Table 3; complete parsimonious-model coefficients, standard errors, and Wald statistics are reported in Table 4.

**Figure 1:**
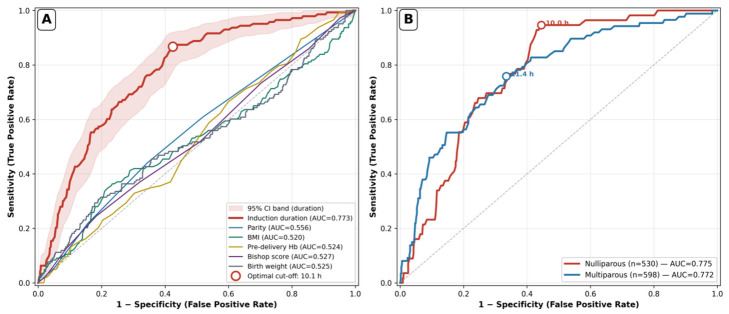
ROC curve analysis for prediction of postpartum haemorrhage. (**A**) Discriminative ability of induction duration (apparent AUC 0.773; 95% CI 0.734–0.809; optimism-corrected AUC 0.773; shaded band: 95% bootstrap CI) compared with parity (AUC 0.556), BMI (AUC 0.520), pre-delivery haemoglobin (AUC 0.524), Bishop score (AUC 0.527), and birth weight (AUC 0.525). Open circle marks the Youden-optimal cut-off of 10.1 h (sensitivity 86.7%, specificity 57.6%). (**B**) Parity-stratified ROC analysis: optimal threshold 10.0 h in nulliparous (AUC 0.775) and 11.4 h in multiparous (AUC 0.772) patients. AUC, area under the receiver operating characteristic curve; CI, confidence interval; PPH, postpartum haemorrhage.

ROC curve analysis for total induction duration is illustrated in Figure 1A. The apparent AUC was 0.773 (95% CI 0.734–0.809), indicating moderate discriminative ability, substantially higher than parity (AUC 0.556), BMI (AUC 0.520), pre-delivery Hb (AUC 0.524), Bishop score (AUC 0.527), or birth weight (AUC 0.525), whose curves are shown for comparison. Bootstrap internal validation (2000 replicates) yielded a mean optimism of <0.001, giving an optimism-corrected AUC of 0.773, confirming minimal overfitting for a single continuous predictor. The Youden-optimal cut-off of 10.1 h yielded sensitivity 86.7% (95% CI 80.2–91.3%), specificity 57.6% (95% CI 54.5–60.6%), PPV 22.9% (95% CI 19.5–26.6%), and NPV 96.8% (95% CI 95.0–97.9%) at the observed cohort prevalence of 12.7%. The positive and negative likelihood ratios were 2.04 and 0.23, respectively—prevalence-independent measures that allow translation of the threshold to settings with different baseline PPH rates. In practical terms, at our cohort prevalence, the high NPV implies that patients delivered within 10.1 h have a greater than 96% probability of not developing PPH in this population, potentially supporting decisions about routine versus heightened postpartum monitoring within established institutional surveillance pathways. Bootstrap-based assessment of threshold stability gave a median cut-off of 10.2 h (IQR 10.0–11.3 h), confirming robustness to sampling variability. The E-value [21] for the duration effect was 1.47 (lower bound 1.41), for parity 1.50, and for emergency caesarean 1.78. An unmeasured confounder would need to be associated with both induction duration and PPH by a relative risk of ≥1.41, above and beyond the measured covariates, to fully explain away the observed association—a magnitude that cannot be ruled out in this retrospective design, given plausible unmeasured factors such as baseline uterine contractility, ethnicity, or socioeconomic status.

**Figure 2:**
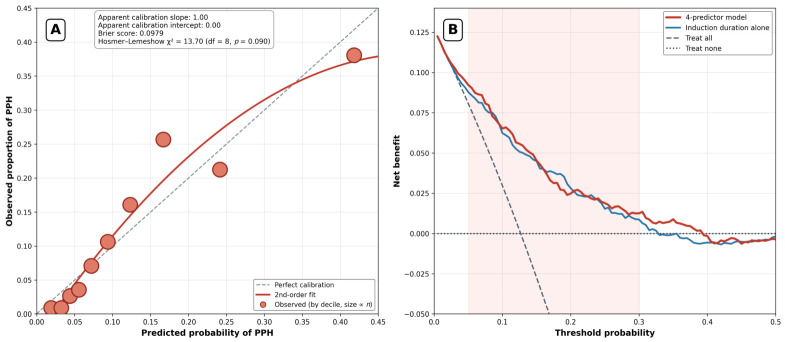
Calibration and decision curve analysis of the parsimonious 4-predictor model (*n* = 1128). (**A**) Decile-based calibration plot. Predicted versus observed probabilities of postpartum haemorrhage, with point size proportional to the number of observations within each decile. The 2nd-order polynomial fit (red curve) tracks the diagonal line of perfect calibration (grey dashed) across the risk spectrum, with modest over-prediction in the highest-risk decile. (**B**) Decision curve analysis comparing the parsimonious 4-predictor model (red) against a single-predictor duration-only model (blue), and the reference strategies of treating all patients (grey dashed) and treating none (black dotted). The shaded region (5–30%) indicates the range of threshold probabilities most likely to be clinically relevant for postpartum haemorrhage risk-stratified intervention. The 4-predictor model demonstrated positive net benefit across the entire clinically relevant range, consistently superior to both reference strategies and to the duration-only model.

Subgroup ROC curves stratified by parity are displayed in Figure 1B; the optimal threshold was 10.0 h in nulliparous patients (AUC 0.775, sensitivity 94.6%) and 11.4 h in multiparous patients (AUC 0.772, sensitivity 75.9%), with nearly identical AUCs confirming cross-parity consistency of the ~10 h regional threshold. Calibration assessment of the final four-predictor parsimonious model showed an apparent calibration slope of 1.00 and intercept of 0.00 (inherent to apparent fit); the Hosmer–Lemeshow goodness-of-fit test was not significant (χ^2^ = 13.70; *p* = 0.090), and the decile-based calibration plot (Figure 2A) demonstrated acceptable agreement between predicted and observed probabilities across the risk spectrum, with modest over-prediction in the highest-risk decile. External validation remains necessary before deployment in populations with different baseline PPH prevalence.

PPH rates and crude ORs by induction agent are shown in Table 5. The oxytocin plus Foley balloon combination carried the highest PPH rate (20.0%; OR 2.44 versus oxytocin monotherapy; 95% CI 1.46–4.09), followed by Foley balloon monotherapy (16.7%; OR 1.95; 95% CI 1.09–3.49) and Propess monotherapy (16.4%; OR 1.92; 95% CI 1.22–3.02). Oxytocin plus Propess did not differ significantly from oxytocin alone (9.6% vs. 9.3%; *p* = 0.928). When induction duration was also included in the model, the apparent Foley effect was attenuated to non-significance (aOR 1.580; *p* = 0.131), suggesting that the higher crude PPH rate associated with Foley-containing regimens may be partly explained by the longer induction durations required for an unfavourable cervix. A multiplicative interaction between induction duration and Foley balloon use showed a borderline trend that did not reach conventional statistical significance (aOR 0.912; *p* = 0.051), with a negative coefficient suggesting that the per-hour risk increment attributable to duration may be modestly attenuated in the Foley balloon subgroup. The directional pattern is consistent with absolute duration—rather than the device itself—being the principal driver of risk; nonetheless, because these subgroup analyses are underpowered, a smaller independent device effect cannot be excluded, and this interpretation is hypothesis-generating.

Calibration of the parsimonious 4-predictor model was satisfactory. Apparent calibration slope was 1.00 and intercept 0.00 (inherent to apparent fit), with a Brier score of 0.098 and a non-significant Hosmer–Lemeshow goodness-of-fit test (χ^2^ = 13.70; df = 8; *p* = 0.090). Bootstrap internal validation (2000 replicates) yielded optimism-corrected calibration slope 0.973, intercept −0.034, and Brier score 0.099, indicating minimal overfitting and stable calibration across resampling. The decile-based calibration plot is presented in Figure 2A. Decision curve analysis (Figure 2B) demonstrated positive net benefit of the parsimonious model across the full range of clinically plausible threshold probabilities (5–30%; shaded region). Because decision curve analysis is defined over threshold probabilities, the net benefit of 0.055 reported here corresponds to a threshold probability equal to the cohort prevalence (12.7%) and should not be interpreted as the net benefit of the 10.1 h duration cut-off itself; at this threshold the “treat all” and “treat none” strategies yield 0.000 and 0.000, respectively. At a threshold of 20%—a value compatible with allocating enhanced third-stage vigilance and team preparedness to higher-risk patients—the model retained a net benefit of 0.025, while a “treat all” strategy carried a substantial negative net benefit (–0.092). The 4-predictor model showed marginal but consistent superiority over the duration-only model across this threshold range, supporting the inclusion of age, parity, and emergency caesarean status as clinically meaningful covariates beyond duration.

**Figure 3:**
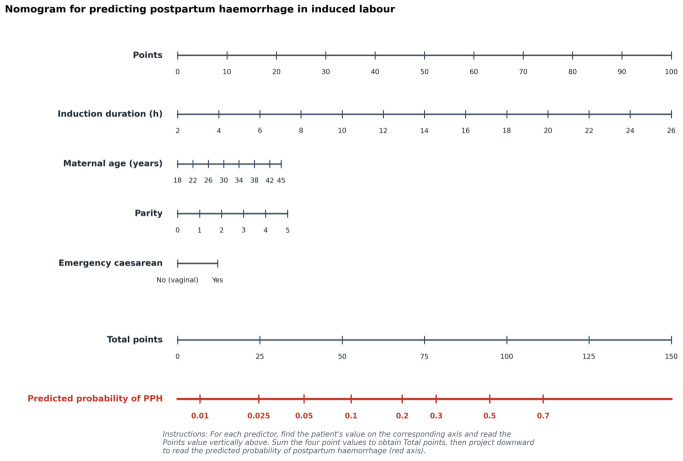
Nomogram for predicting postpartum haemorrhage in induced labour. Probability-scaled nomogram derived from the parsimonious 4-predictor logistic regression model (induction duration, maternal age, parity, emergency caesarean delivery; *n* = 1128). For each predictor, the patient’s value is located on the corresponding axis and the points value read vertically above on the Points scale. The four point values are summed to obtain Total points, which is then projected downward to read the predicted probability of postpartum haemorrhage on the red probability axis. Axis lengths reflect each predictor’s relative contribution to the linear predictor: induction duration is the dominant determinant, while emergency caesarean carries the smallest single-variable weight. Intended clinical use: the tool can be applied (i) at admission or during early induction, as a partial dynamic estimate based on age, parity and evolving induction duration, to inform shared decision-making about the threshold for closer reassessment and intensified monitoring; and (ii) immediately after delivery, when delivery mode is known and all four predictors are available, to guide the intensity of third-stage prophylaxis and early postpartum surveillance. The complete four-predictor probability estimate is therefore obtainable only after delivery mode has been ascertained. Worked example: a 32-year-old nulliparous woman who underwent a 14 h induction culminating in emergency caesarean accumulates approximately 69 total points, corresponding to a predicted PPH probability of ≈0.21—a risk level at which enhanced readiness, structured reassessment, and active third-stage vigilance may be warranted. External validation is required before incorporation into institutional protocols.

To facilitate translation of the parsimonious 4-predictor model into clinical practice, a nomogram was constructed (Figure 3). Each predictor is mapped to a points scale anchored such that the variable with the largest contribution to the linear predictor (induction duration, spanning approximately 100 points across its observed range of 2–26 h) corresponds to a 100-point reference. Maternal age contributed up to ≈21 points across 18–45 years, parity up to ≈22 points across 0–5 deliveries, and emergency caesarean delivery contributed ≈8 points (binary). For a representative example—a 32-year-old nulliparous woman who underwent a 14 h induction subsequently culminating in emergency caesarean—the nomogram yields approximately 69 total points, corresponding to a predicted PPH probability of 0.21, in agreement with the direct model output (0.206). The nomogram is intended for use as a clinical decision-support tool pending external validation.

Sensitivity analyses and subgroup findings are summarised in Table 6 and Table 7, respectively. When the analysis was restricted to vaginal deliveries (*n* = 789; PPH rate 10.9%), induction duration remained a strong independent predictor (aOR 1.272; 95% CI 1.201–1.348; *p* < 0.001), closely mirroring the primary model. The corresponding ROC AUC was 0.798 with an identical 10.1 h optimal threshold. Restricting the outcome to severe PPH (*n* = 43; 3.8%) preserved the primacy of induction duration (aOR 1.194; 95% CI 1.119–1.274; *p* < 0.001), while parity (aOR 1.026; *p* = 0.838) and emergency caesarean (aOR 1.681; *p* = 0.109) lost statistical significance—a finding most consistent with reduced statistical power in this smaller event cohort (EPV = 10.75) rather than absence of biological effect, given that point estimates for all four predictors remained directionally consistent. The ROC-derived cut-off for severe PPH was 10.2 h (AUC 0.760; sensitivity 90.7%). Crucially, restricting analysis to oxytocin monotherapy (*n* = 571; 53 PPH events) preserved and indeed strengthened the duration signal (aOR 1.335 per hour; 95% CI 1.238–1.439; *p* < 0.001; AUC 0.814; identical 10.1 h cut-off), supporting that the duration-PPH association is not an artefact of co-administration with mechanical ripening agents. In subgroup analyses (Table 7), the optimal thresholds were 10.0 h in nulliparous and 11.4 h in multiparous patients, 13.5 h in obese patients (BMI ≥ 30) reflecting both higher baseline PPH rates (18.7%) and a right-shifted duration distribution, and comparable 10.0–10.3 h in both anaemic (pre-Hb < 11 g/dL; AUC 0.787) and non-anaemic (AUC 0.770) subgroups. In a post hoc sensitivity model that additionally included epidural analgesia as a covariate, the adjusted odds ratio for induction duration changed only marginally (from 1.243 to 1.248 per hour; +0.4%), confirming that the principal duration–PPH association is not materially confounded by epidural exposure; epidural analgesia itself was associated with PPH (aOR 2.482, 95% CI 1.274–4.836; *p* = 0.008), consistent with mediation by prolonged or operative labour; and because epidural use plausibly marks longer and more complex labour, this association is hypothesised rather than established and cannot be confirmed from cross-sectional data.

To examine whether the duration–PPH relationship varied with baseline haemoglobin reserve, the cohort was stratified by pre-delivery Hb tertiles (Supplementary Figure S2). Induction duration remained an independent predictor of PPH within each tertile after multivariable adjustment (lowest tertile, Hb 8.1–11.3 g/dL: aOR 1.238 per hour, 95% CI 1.142–1.342, *p* < 0.001; middle tertile, Hb 11.4–12.5: aOR 1.195, 95% CI 1.120–1.275, *p* < 0.001; highest tertile, Hb 12.6–15.0: aOR 1.359, 95% CI 1.243–1.485, *p* < 0.001). Optimal Youden thresholds were 10.2 h in the lowest tertile (AUC 0.758), 10.0 h in the middle tertile (AUC 0.746), and 12.7 h in the highest tertile (AUC 0.818), supporting threshold stability across the lower two-thirds of the haemoglobin distribution. The formal test for duration × Hb-tertile interaction approached but did not reach statistical significance (likelihood-ratio χ^2^ = 5.20, df = 2, *p* = 0.074), suggesting a borderline trend toward effect modification rather than a definitive interaction.

## 4. Discussion

In this analysis of 1128 consecutive induced labours at a high-volume Turkish tertiary centre, we report three principal findings. First, PPH—defined by an objective haemoglobin-drop criterion—occurred in 12.7% of patients, with severe PPH in 3.8%, broadly consistent with published rates in induced cohorts when the outcome is captured by objective laboratory rather than volumetric criteria. Second, total induction duration emerged as a robust and clinically meaningful independent predictor of PPH, surviving adjustment for age, parity, emergency caesarean, Foley balloon use, BMI, birth weight, Bishop score, and pre-delivery haemoglobin, and yielding an optimism-corrected AUC of 0.773—reflecting moderate but clinically informative discrimination—that substantially outperformed all other candidate markers. Third, the Youden-optimal threshold of 10.1 h was robust across parity, delivery-mode, and—most importantly—oxytocin-only subgroups, supporting that the association is not an artefact of co-administration with mechanical ripening agents.

Our PPH incidence of 12.7% sits within the 10–15% range typically reported in obstetric cohorts using comparable Hb-based definitions [18,19] and exceeds population-wide visually estimated rates, as expected given the induced-only case mix, the 30% emergency caesarean rate characteristic of tertiary referral practice, and the objective Hb-drop criterion. The use of a haemoglobin-drop threshold in place of the ACOG/FIGO-preferred volumetric criterion (cumulative blood loss ≥ 1000 mL or haemodynamic instability) warrants explicit discussion. On one hand, the Hb-drop approach offers objectivity and reproducibility that visually estimated blood loss cannot match, especially in retrospective cohorts where quantitative blood loss was not uniformly implemented. On the other hand, it introduces two known measurement artefacts: perioperative crystalloid infusion may dilute postpartum haemoglobin and inflate the apparent drop, whereas baseline anaemia compresses the achievable absolute drop and may under-detect clinically important bleeding. Both of these biases are non-differential with respect to induction duration and therefore tend to attenuate the observed association, rendering our effect-size estimates conservative rather than inflated. Nonetheless, this definitional choice means that our incidence and severity figures are not directly comparable with cohorts using volumetric definitions, and externally valid integration into ACOG/FIGO-aligned protocols mandates prospective studies that apply quantitative blood loss measurement in parallel with haemoglobin tracking before any clinical application. Because incidence and severity estimates obtained under a haemoglobin-drop definition are not interchangeable with volumetric ones, our incidence (12.7%) and severity figures are not directly comparable with volumetrically defined cohorts, and clinical adoption of the 10.1 h threshold would require recalibration in any setting using those definitions.

The independent association between induction duration and PPH (aOR 1.243 per hour) is biologically consistent with several candidate mechanisms that would operate only if the relationship is at least partly causal: cumulative oxytocin-receptor desensitisation [8,9], myometrial glycogen depletion and oxidative stress [23], and the downstream consequences of dysfunctional labour patterns that often accompany prolonged induction. These mechanisms are hypothesised rather than demonstrated by the present observational design. Grotegut et al. [3] first demonstrated that longer oxytocin exposure—whether measured as cumulative dose or infusion duration—is associated with uterine atony-related PPH; Belghiti et al. [24] corroborated this in a large case–control series. Our ROC analysis translates this continuous relationship into a practical 10.1 h threshold that should be interpreted not as marking a specific biological transition point, but rather as an empirically derived inflection in the duration–risk curve. Bootstrap stability (median 10.2 h; IQR 10.0–11.3 h), near-identity in nulliparous (10.0 h) and vaginal-only (10.1 h) subgroups, and preservation in the oxytocin-only subgroup (10.1 h; AUC 0.814) suggest physiological regularity rather than a population-specific artefact. The slightly later thresholds in multiparous (11.4 h) and obese (13.5 h) subgroups likely reflect right-shifted duration distributions rather than a fundamentally different risk physiology. The NPV of 96.8% at our cohort prevalence supports routine postpartum care for patients delivered within this window, while those beyond it warrant heightened haemostatic vigilance—an approach consistent with guideline-based recommendations on haemorrhage preparedness for patients with elevated PPH risk [9].

Given the retrospective, single-centre design, this threshold is best regarded as a candidate benchmark rather than a definitive clinical decision rule. The E-value [21] for the duration effect (1.47; lower bound 1.41) indicates that an unmeasured confounder would need to be associated with both induction duration and PPH by an adjusted relative risk of at least 1.41 to fully explain away the observed association—a plausible but not trivial magnitude. While this does not exclude residual confounding, an unmeasured confounder of the required magnitude (RR ≈ 1.41) is plausible in observational obstetric data and cannot be excluded; weaker confounding, however, would leave a substantive residual duration–PPH association intact. The question of reverse causality deserves particular emphasis: prolonged induction may not itself cause PPH, but may instead serve as an early clinical manifestation of intrinsically dysfunctional myometrial contractility, which in turn predisposes to postpartum atony and haemorrhage. Under this alternative framing, induction duration would act as a marker of an underlying myometrial phenotype rather than as an independent upstream risk factor. Although our data cannot arbitrate between a causal (cumulative exposure) and a marker-of-dysfunction interpretation—and the modest E-value of 1.47 leaves a reverse-causality interpretation entirely plausible—three observations are at least compatible with a partial causal contribution, without establishing one: the aOR for induction duration was remarkably stable across four different modelling strategies (vaginal-only 1.272; severe-PPH 1.194; oxytocin-only 1.335; primary 1.243); the oxytocin-only subgroup—where the duration signal represents the most direct measurable surrogate of cumulative oxytocin-receptor exposure—showed the strongest effect (AUC 0.814); and the E-value of 1.47 quantifies the strength of unmeasured confounding (including a latent contractility phenotype) that would be required to fully explain away the observed effect—a magnitude that is plausible in observational obstetric data and cannot be definitively excluded, although weaker confounding would leave a residual duration–PPH association intact. Even under a purely marker interpretation, however, the clinical utility of the 10.1 h threshold for risk stratification remains: a patient whose induction has exceeded this window has, by observation, entered a higher-risk state irrespective of whether that state reflects an underlying biological vulnerability or cumulative iatrogenic exposure. Prospective external validation in at least two to three independent centres—ideally capturing cumulative oxytocin dose and baseline contractility correlates where feasible—is required before integration into routine clinical protocols.

Foley-containing regimens, although associated with higher unadjusted PPH rates (16.7% for Foley alone, 20.0% for oxytocin plus Foley, versus 9.3% for oxytocin alone), did not remain significant after adjustment (aOR 1.580; *p* = 0.131). This suggests that the apparent haemorrhagic penalty of mechanical ripening may be largely explained by the longer total induction intervals that mechanical methods impose on patients with an unfavourable cervix. Plausible device-specific mechanisms—local prostaglandin E2 release and pro-inflammatory cytokine upregulation [12,25]—remain possible but appear quantitatively smaller than the time-dependent effect once duration is controlled. Propess-containing regimens similarly showed no significant adjusted association (aOR 1.411; *p* = 0.253), with oxytocin plus Propess yielding a PPH rate (9.6%) essentially identical to oxytocin alone (9.3%). The null adjusted findings for both mechanical and prostaglandin-based agents should be interpreted cautiously given limited subgroup power [26]; propensity-score replication in external datasets would help confirm these interpretations. The borderline duration × Foley interaction (aOR 0.912; *p* = 0.051) did not reach significance and is therefore hypothesis-generating; its direction is consistent with absolute duration—rather than the device per se—being the principal driver of risk, but a smaller independent device effect cannot be excluded given limited power.

Multiparity as an independent predictor (aOR 1.262 per unit increase) is consistent with progressive myometrial remodelling with each successive pregnancy, reducing the contractile reserve available for third-stage haemostasis. Parity was modelled as a continuous linear term; grand multiparity (≥5 deliveries) likely confers disproportionately higher risk, though subgroup analyses specifically targeting grand multiparous women were not feasible given cell sizes [27]. This finding aligns with large cohort data from Ende et al. [27] and Kramer et al. [28]. Maternal age also emerged as a small but statistically significant independent predictor (aOR 1.041 per year), compatible with age-related changes in myometrial contractility described in prior work. In contrast, elevated BMI did not emerge as an independent predictor after full adjustment (aOR 1.005 per kg/m^2^; *p* = 0.831) despite a numerically higher PPH rate in obese women (18.7%). Inspection of the categorical distribution in Table 1 suggests a non-linear relationship with elevated risk at both BMI extremes (21.7% in underweight, 18.7% in obese, versus 8.8–12.0% at intermediate categories) that was not fully captured by linear parameterization. This pattern is consistent with BMI acting as a proxy for longer induction intervals and operative delivery rates at the extremes [10,29], rather than as an independent linear driver. Similarly, higher birth weight (aOR 1.192 per 500 g; *p* = 0.145) approached but did not reach statistical significance; its direction of effect and the well-established mechanism of overdistension-related atony [30] warrant spline-based or fractional-polynomial modelling in larger cohorts. The initial Bishop score reached borderline statistical significance (aOR 1.135 per unit; *p* = 0.050), suggesting that an unfavourable cervix may impose a small independent contribution to PPH risk—potentially mediated by the longer induction trajectory required to achieve adequate cervical ripening; however, given the borderline *p*-value and the partial overlap with induction duration, this association should be regarded as suggestive rather than definitive and warrants confirmation in independent cohorts.

Emergency caesarean delivery (aOR 1.527) augments risk through its larger uterine incision surface, anaesthetic haemodynamic perturbation, and frequent co-occurrence with labour dystocia. Its loss of significance in the severe-PPH sensitivity analysis (aOR 1.681; *p* = 0.109) most plausibly reflects reduced statistical power with only 43 events rather than absence of biological effect: the point estimate remained directionally consistent and, if anything, larger than in the primary model. This highlights a general feature of rare-outcome analyses in our dataset and reinforces the importance of the primary ≥ 2 g/dL outcome for adequately powered inference.

Two further observations merit comment. Active labour duration was numerically shorter in the PPH group (median 4.8 vs. 5.3 h; *p* = 0.418) among vaginal deliveries only, likely reflecting truncation by the higher emergency caesarean rate in the PPH group; total induction duration was nevertheless substantially longer (14.3 vs. 9.9 h), consistent with prolonged pre-active-labour ripening. Manual placental delivery was performed in 8.9% of cases and carried a higher PPH rate (22.0% vs. 11.8%; *p* = 0.005); as both a marker of incomplete third-stage separation and a cause of additional endometrial trauma [31], manual removal was considered a downstream mediator rather than an upstream risk factor. Among vaginal deliveries, third- or fourth-degree perineal laceration (6.6%) was associated with a higher PPH rate (23.1% vs. 10.0%; *p* = 0.007), through direct soft-tissue blood loss compounding concurrent uterine atony [32]. Apgar scores did not differ between groups (both median 9, *p* > 0.4), indicating no measurable neonatal compromise within our induced cohort—a reassuring finding that likely reflects prompt clinical intervention, although umbilical cord gases and NICU admission data were not available for more sensitive ascertainment.

## 5. Clinical Application

Before considering the applications discussed below, it must be emphasised that they remain contingent on prospective external validation: because these findings derive from a single tertiary centre with a 30.1% emergency caesarean rate, a TOLAC-naive population, and a specific low-dose oxytocin protocol, the 10.1 h threshold is a hypothesis-generating benchmark requiring confirmation in independent populations and is not yet ready for clinical use. Importantly, the clinical value of induction duration lies in its real-time availability, distinguishing it from most static antepartum risk factors and enabling dynamic intrapartum risk stratification. Our findings may inform future development of time-based risk-stratification approaches for induced labour, particularly as such an approach could be considered without requiring new technologies or laboratory tests. At the 10.1 h threshold, the high negative predictive value (96.8%) suggests that, subject to prospective external validation, women who deliver within this window might be considered for a standard third-stage and postpartum observation pathway, whereas those crossing the threshold might warrant consideration of enhanced preparedness measures within existing institutional protocols and without replacing established, guideline-based risk-assessment frameworks, such as additional large-bore intravenous access, immediate availability of a type-and-screen specimen, anticipatory preparation of second-line uterotonics (methylergometrine, carboprost, or misoprostol in accordance with local protocols and contraindications), proactive engagement of the anaesthesia and blood-bank teams, and continuous quantitative blood loss monitoring during and after delivery. Parity-stratified thresholds (10.0 h in nulliparous; 11.4 h in multiparous women) may be more appropriate where protocol granularity permits. Importantly, any future time-based threshold would be intended to complement, not replace, established antepartum and intrapartum risk-assessment tools such as those of the California Maternal Quality Care Collaborative and the Association of Women’s Health, Obstetric and Neonatal Nurses; a patient flagged as high-risk by any pre-existing criterion should continue to receive maximal preparation regardless of elapsed induction time. Operationally, the threshold could be conceptualised as a labour-ward checkpoint—at approximately the 10 h mark, a structured re-evaluation of progress, cumulative oxytocin exposure, and delivery plan may prompt intensified preparedness and closer monitoring rather than any specific intervention. Before any such integration, however, external validation in independent multicentre cohorts—ideally with concurrent quantitative blood loss capture—is essential, and clinicians should recognise that the 10.1 h threshold is an empirically derived inflection point rather than a biologically immutable cut-off. Importantly, this threshold should not be interpreted as a trigger for intervention, but rather as a clinical prompt for heightened awareness and preparedness. Accordingly, the threshold is best viewed as a dynamic risk marker within the clinical context rather than a fixed decision boundary.

Beyond conventional discrimination metrics, decision curve analysis confirmed that the parsimonious 4-predictor model provides positive net clinical benefit across the range of threshold probabilities most likely to inform real-world risk-stratified intervention (5–30%), with consistent superiority over both “treat all” and duration-only strategies in this range. The accompanying nomogram (Figure 3) translates the model into a bedside-usable graphical tool, allowing clinicians to read individualised PPH probabilities directly from four routinely documented variables. Our nomogram contributes to a small but rapidly growing literature on probability-scaled nomograms for PPH risk stratification: existing tools have been developed for general vaginal delivery cohorts [33] and for high-risk subpopulations such as caesarean delivery in twin pregnancies [34]. The present model addresses a complementary clinical niche—the broad induced-labour cohort at term—and uniquely positions induction duration as the dominant modifiable predictor, distinguishing it from population-specific tools that emphasise non-modifiable demographic or obstetric risk factors. While external validation remains necessary before institutional adoption, this format facilitates incorporation into intrapartum risk-assessment workflows in a manner that simple threshold-based rules do not.

Stratified analysis by pre-delivery haemoglobin tertiles refined our understanding of the duration–PPH relationship. While the dichotomous anaemic-versus-non-anaemic comparison demonstrated robustness of the 10.1 h threshold, tertile-level analysis revealed a more nuanced pattern: the threshold remained essentially identical (10.0–10.2 h) in the lowest two tertiles—covering Hb values up to 12.5 g/dL—whereas the highest tertile (Hb 12.6–15.0 g/dL) yielded a longer threshold of 12.7 h alongside a numerically higher discriminative AUC (0.818). The formal interaction test yielded a borderline result (*p* = 0.074), precluding strong inference, but the directional pattern is consistent with a haemoglobin-reserve hypothesis whereby women with higher pre-delivery red-cell reserves may tolerate prolonged induction with comparatively less haemorrhagic vulnerability. Because the formal interaction was not significant, the principal 10.1 h threshold should be retained for general use; the tertile-specific findings should be regarded as hypothesis-generating and warrant prospective confirmation.

## 6. Strengths and Limitations

Several strengths distinguish this study. With 143 PPH events among 1128 cases, the events-per-variable ratio (14.3 for the full model; 35.8 for the parsimonious model) comfortably exceeds both the classical minimum of 10 and the shrinkage-based criteria proposed by Riley and colleagues [20]. The duration estimate was stable across all four pre-specified models (aOR 1.194–1.335), and 2000-replicate bootstrap validation (Harrell optimism correction) confirmed negligible optimism (optimism-corrected AUC 0.773). Robustness to unmeasured confounding was quantified via E-values [21], and the oxytocin-only subgroup (aOR 1.335; AUC 0.814) provides the closest available approximation to a pure duration–PPH relationship free from confounding by mechanical ripening. The single-centre design, the objective Hb-drop outcome, and dual-investigator data extraction further reduce protocol, observer, and data-quality variation. To our knowledge, this is the first ROC-validated, bootstrap-stable, parity-stratified induction-duration threshold reported from a Turkish tertiary centre.

Several limitations deserve explicit acknowledgement. First, the retrospective design precludes causal inference; residual and unmeasured confounding—including uterine anomalies, granular oxytocin dose-escalation records, individual clinician operative thresholds, and perioperative intravenous fluid volumes—cannot be fully excluded. Second, as detailed in the Discussion, reverse causality cannot be excluded: induction duration may partly act as a marker of an underlying myometrial phenotype rather than a purely causal exposure. Third, our haemoglobin-drop definition of PPH departs from the ACOG/FIGO volumetric standard (≥1000 mL) and carries two measurement artefacts: perioperative crystalloid infusion may dilute postpartum haemoglobin and inflate the apparent drop—a concern most relevant to the 30.1% of patients delivered by caesarean—whereas baseline anaemia (present in 23.7%; *n* = 267) compresses the achievable absolute drop and may under-detect clinically important bleeding. Both biases are non-differential with respect to induction duration and therefore tend to bias estimates towards the null. Intravenous fluid volumes were not systematically recorded, precluding a fluid-adjusted analysis. External validation against quantitative blood loss (QBL) is therefore mandatory before any clinical use, and adoption within ACOG/FIGO-aligned protocols will require prospective studies that apply QBL in parallel with haemoglobin tracking [35]. Fourth, cumulative oxytocin dose—the most proximate mechanistic correlate of receptor desensitisation—was not available; although the oxytocin-only subgroup partly addresses this, formal dose–response modelling awaits prospective data. Fifth, continuous covariates (BMI, birth weight, parity) were modelled as linear terms; the non-linear BMI pattern observed in Table 1 warrants spline or fractional-polynomial modelling in future work. Sixth, the severe-PPH sensitivity analysis was underpowered (43 events), and loss of statistical significance for parity and emergency caesarean in that analysis should be interpreted as a power issue rather than as biological evidence against these predictors. Seventh, the high emergency caesarean rate (30.1%) reflects tertiary referral concentration and limits generalisability to lower-acuity settings. Eighth, single-centre, single-calendar-year (2025) design ensures protocol homogeneity but limits temporal and geographic generalisability. Finally, although internal validation was performed using 2000-replicate bootstrap resampling with Harrell optimism correction, external validation in independent multicentre populations is required to confirm generalisability, and the 10.1 h threshold should not be incorporated into institutional protocols until such validation is available; the present findings should therefore be interpreted within the context of a single-centre retrospective design. Epidural analgesia was also associated with PPH in our cohort; because epidural use plausibly marks longer and more complex labour, this association is most consistent with mediation rather than a direct haemorrhagic effect and cannot be resolved with cross-sectional data [36]. Ethnicity and socioeconomic deprivation, recognised determinants of PPH risk in high-income settings [37], were likewise not captured in our dataset, reinforcing the possibility of residual confounding noted above.

## 7. Conclusions

Total induction duration emerged as a robust and clinically meaningful independent predictor of laboratory-defined PPH in this single-centre retrospective cohort, with a bootstrap-stable 10.1 h threshold demonstrating moderate but clinically informative discrimination (optimism-corrected AUC 0.773). These findings suggest that prolonged induction is a clinically useful marker of haemorrhagic risk; whether the relationship is causal or partly reflects an underlying myometrial phenotype awaits prospective confirmation. The apparent risk signal from Foley balloon use may be partly explained by the longer durations it entails, although a smaller independent device effect cannot be definitively excluded. Given the observational design and single-centre setting, these results should be interpreted as hypothesis-generating rather than as definitive evidence for immediate practice change; accordingly, prospective, multicentre studies are warranted to externally validate the identified threshold and to determine whether time-based risk stratification can improve clinical outcomes in induced labour.

Beyond a single threshold, the parsimonious 4-predictor model demonstrated favourable calibration and positive net clinical benefit on decision curve analysis across the range of threshold probabilities most likely to inform real-world risk-stratified intervention; an accompanying nomogram is provided to facilitate exploratory bedside application pending external validation. Tertile-stratified analysis confirmed that the 10.1 h threshold remained essentially identical across the lower two-thirds of the pre-delivery haemoglobin distribution, with a borderline trend toward effect modification at the highest tertile that warrants further prospective study.

## Figures and Tables

**Table 1:** Baseline demographic and clinical characteristics stratified by PPH status.

Variable	All (*n* = 1128)	PPH (*n* = 143; 12.7%)	Non-PPH (*n* = 985; 87.3%)	*p* Value
Age (years)	28.0 ± 5.0	28.9 ± 5.2	27.9 ± 5.0	0.038
BMI (kg/m^2^) ‡	26.8 (23.5–29.7)	27.4 (23.2–31.1)	26.7 (23.5–29.4)	0.439
Underweight (<18.5)	46 (4.1%)	10 (7.0%)	36 (3.7%)	
Normal (18.5–24.9)	349 (30.9%)	42 (29.4%)	307 (31.2%)	
Overweight (25.0–29.9)	465 (41.2%)	41 (28.7%)	424 (43.0%)	
Obese (≥30.0)	268 (23.8%)	50 (35.0%)	218 (22.1%)	
Gravida ‡	2.0 (1.0–3.0)	2.0 (1.0–3.0)	2.0 (1.0–3.0)	0.043
Parity ‡	1.0 (0.0–2.0)	1.0 (0.0–2.0)	1.0 (0.0–2.0)	0.021
Nulliparous	530 (47.0%)	56 (39.2%)	474 (48.1%)	
Multiparous	598 (53.0%)	87 (60.8%)	511 (51.9%)	
Gestational age at induction (wks)	40.2 ± 1.5	40.2 ± 1.5	40.2 ± 1.5	0.907
Initial Bishop score	3.7 ± 2.0	3.9 ± 2.0	3.6 ± 2.0	0.224
Pre-delivery Hb (g/dL)	11.91 ± 1.27	12.02 ± 1.15	11.89 ± 1.28	0.263
Baseline anaemia (Hb < 11 g/dL)	267 (23.7%)	29 (20.3%)	238 (24.2%)	0.318
Post-delivery Hb (g/dL)	10.75 ± 1.48	9.20 ± 1.35	10.98 ± 1.35	<0.001
Hb drop (g/dL) ‡	1.0 (0.6–1.4)	2.6 (2.3–3.2)	0.9 (0.6–1.2)	<0.001
Total induction duration (h)	10.5 ± 4.5	14.3 ± 4.1	9.9 ± 4.3	<0.001
Active labour duration (h), vaginal only ‡	5.2 (3.5–6.8)	4.8 (3.4–6.6)	5.3 (3.5–6.8)	0.418
Second-stage duration (min), vaginal only ‡	32 (19–49)	28 (18–47)	33 (19–49)	0.373
Birth weight (g) ‡	3297 (3017–3588)	3337 (2977–3692)	3296 (3026–3573)	0.334

Data are mean ± SD or ‡ median (Q1–Q3). Student’s *t*-test for continuous variables with approximately normal distributions (skewness < 0.5, kurtosis < 1); Mann–Whitney U test for non-normal continuous variables; chi-square or Fisher’s exact test for categorical variables. Active labour and second-stage durations are restricted to vaginal deliveries (*n* = 789) to avoid truncation bias from emergency caesareans performed before labour completion. BMI, body mass index; BPP, biophysical profile; Hb, haemoglobin.

**Table 2:** Obstetric and induction-related parameters stratified by PPH status.

Variable	PPH (*n* = 143)	Non-PPH (*n* = 985)	*p* Value
Mode of delivery			0.008
Vaginal (NSD)	86 (60.1%)	703 (71.4%)	
Emergency caesarean	57 (39.9%)	282 (28.6%)	
Induction agent			0.002
Oxytocin (reference)	53 (37.1%)	518 (52.6%)	
Propess (dinoprostone insert)	37 (25.9%)	188 (19.1%)	
Foley balloon	18 (12.6%)	90 (9.1%)	
Oxytocin + Propess	9 (6.3%)	85 (8.6%)	
Oxytocin + Foley balloon	26 (18.2%)	104 (10.6%)	
Induction indication			0.425
Post-term pregnancy (≥41 wks)	75 (52.4%)	435 (44.2%)	
PROM	35 (24.5%)	305 (31.0%)	
Oligohydramnios	16 (11.2%)	114 (11.6%)	
Abnormal BPP	14 (9.8%)	106 (10.8%)	
Gestational diabetes (diet/metformin)	3 (2.1%)	25 (2.5%)	
Episiotomy (vaginal deliveries only)	56/86 (65.1%)	468/703 (66.6%)	0.882
Epidural analgesia	15 (10.5%)	54 (5.5%)	0.032
Manual placental delivery	22 (15.4%)	78 (7.9%)	0.005
Additional uterotonics required	95 (66.4%)	54 (5.5%)	<0.001
Methylergometrine monotherapy	53 (37.1%)	28 (2.8%)	
Misoprostol monotherapy	16 (11.2%)	16 (1.6%)	
Carbetocin	18 (12.6%)	9 (0.9%)	
Methylergometrine + Misoprostol	8 (5.6%)	1 (0.1%)	
Blood transfusion	21 (14.7%)	0 (0.0%)	<0.001
ICU transfer	6 (4.2%)	0 (0.0%)	<0.001

Chi-square test or Fisher’s exact test for all categorical comparisons. Additional uterotonics were administered therapeutically at the clinician’s discretion and were not part of the primary PPH definition. BPP, biophysical profile; ICU, intensive care unit; NSD, normal spontaneous delivery; PROM, premature rupture of membranes.

**Table 3:** Univariable and full multivariable (10-Predictor) logistic regression analysis for PPH.

Variable	Crude OR	95% CI	p (uni)	Adjusted OR	95% CI	p (adj)
Induction duration (per 1 h)	1.234	1.183–1.287	<0.001	1.231	1.175–1.290	<0.001
Age (per year)	1.038	1.002–1.074	0.038	1.042	1.003–1.083	0.036
Parity (per unit)	1.161	1.017–1.324	0.027	1.264	1.090–1.466	0.002
Emergency caesarean (vs. vaginal)	1.652	1.150–2.373	0.007	1.541	1.037–2.291	0.032
Foley-containing agent (vs. oxytocin)	1.812	1.229–2.672	0.003	1.580	0.872–2.863	0.131
Propess-containing agent (vs. oxytocin)	1.237	0.848–1.804	0.270	1.411	0.782–2.546	0.253
BMI (per 1 kg/m^2^)	1.013	0.975–1.053	0.505	1.005	0.963–1.048	0.831
Birth weight (per 500 g)	1.161	0.935–1.441	0.177	1.192	0.941–1.509	0.145
Pre-delivery Hb (per 1 g/dL)	1.083	0.942–1.244	0.263	1.113	0.952–1.302	0.180
Initial Bishop score (per 1-point)	1.056	0.967–1.154	0.224	1.135	1.000–1.289	0.050
Epidural analgesia (yes vs. no)	2.020	1.108–3.686	0.022	—	—	—
Manual placental delivery (yes vs. no)	2.114	1.270–3.520	0.004	—	—	—

OR, odds ratio; CI, confidence interval. Foley-containing agent: Foley balloon or Oxytocin + Foley; Propess-containing agent: Propess or Oxytocin + Propess (reference: oxytocin monotherapy). Adjusted ORs reported in this table correspond to the full 10-predictor model (induction duration, age, parity, emergency caesarean, Foley-containing agent, Propess-containing agent, BMI, birth weight, pre-delivery Hb, and Bishop score). For reference, in this full model the induction-duration aOR is 1.231 (95% CI 1.175–1.290). The parsimonious 4-predictor model (duration + age + parity + emergency caesarean)—used as the reference model throughout the abstract and subsequent analyses—yields slightly different point estimates (duration aOR 1.243, 95% CI 1.191–1.298; age aOR 1.041, 95% CI 1.003–1.082; parity aOR 1.262, 95% CI 1.090–1.460; emergency caesarean aOR 1.527, 95% CI 1.031–2.261), as detailed in Table 4. Epidural analgesia and manual placental delivery were significant univariable predictors but were considered downstream mediators on pre-specified biological grounds (see Discussion) and not included in the adjusted model. All mean-centred VIFs < 1.01 (tolerance > 0.99); Nagelkerke R^2^ = 0.217 (full model) and 0.205 (parsimonious four-predictor model).

**Table 4:** Complete parsimonious four-predictor multivariable logistic regression model—the model used in the abstract and throughout the manuscript.

Predictor	β (SE)	Wald χ^2^	*p* Value	aOR	95% CI	E-Value
Intercept	−6.1146 (0.6692)	83.49	<0.001	—	—	—
Induction duration (per 1 h)	0.2175 (0.0219)	98.27	<0.001	1.243	1.191–1.298	1.47 (1.41)
Maternal age (per 1 year)	0.0406 (0.0194)	4.37	0.037	1.041	1.003–1.082	1.24 (1.06)
Parity (per 1 delivery)	0.2324 (0.0745)	9.74	0.002	1.262	1.090–1.460	1.50 (1.31)
Emergency caesarean (vs. vaginal)	0.4231 (0.2004)	4.46	0.035	1.527	1.031–2.261	1.78 (1.11)

Linear predictor: logit(PPH) = −6.115 + 0.218 × (duration in hours) + 0.041 × (age in years) + 0.232 × (parity) + 0.423 × (emergency caesarean: 1 if yes, 0 if no). Model log-likelihood = −363.76; null log-likelihood = −428.87; Likelihood Ratio χ^2^ = 130.22 (df = 4, *p* < 0.001). McFadden pseudo-R^2^ = 0.152; Nagelkerke R^2^ = 0.205; Brier score = 0.098. Bootstrap optimism-corrected c-statistic = 0.775 (2000 replicates). All mean-centred variance inflation factors < 1.01; tolerance values > 0.99, confirming absence of multicollinearity. E-values computed per VanderWeele–Ding (2017), presented as point estimate (CI-based lower bound). aOR, adjusted odds ratio; CI, confidence interval; SE, standard error.

**Table 5:** PPH Incidence and crude odds ratios by induction agent.

Agent	*n*	PPH *n* (%)	Crude OR	95% CI	*p*
Oxytocin (reference)	571	53 (9.3%)	1.00 (ref)	—	—
Propess	225	37 (16.4%)	1.92	1.22–3.02	0.005
Foley balloon	108	18 (16.7%)	1.95	1.09–3.49	0.023
Oxytocin + Propess	94	9 (9.6%)	1.03	0.49–2.18	0.928
Oxytocin + Foley balloon	130	26 (20.0%)	2.44	1.46–4.09	0.001

Reference group: oxytocin monotherapy. Overall chi-square test for agent: χ^2^ = 17.5; *p* = 0.002.

**Table 6:** Sensitivity analyses of the induction-duration association.

Analysis	N	PPH *n* (%)	aOR Duration	95% CI	*p*	Notes
Primary (full cohort)	1128	143 (12.7%)	1.243	1.191–1.298	<0.001	Reference model
Vaginal deliveries only	789	86 (10.9%)	1.272	1.201–1.348	<0.001	AUC 0.798; cut-off 10.1 h
Severe PPH (Hb drop ≥ 3 g/dL)	1128	43 (3.8%)	1.194	1.119–1.274	<0.001	AUC 0.760; cut-off 10.2 h
Oxytocin monotherapy only	571	53 (9.3%)	1.335	1.238–1.439	<0.001	AUC 0.814; cut-off 10.1 h

aOR for induction duration estimated from multivariable logistic regression within each subsample. Primary model adjusted for age, parity, and emergency caesarean. Vaginal-only model adjusted for age, parity, and Foley-containing agent (emergency caesarean is by definition absent in this subgroup). Severe-PPH and oxytocin-only models adjusted for age, parity, and emergency caesarean. aOR, adjusted odds ratio; AUC, area under the ROC curve; CI, confidence interval; PPH, postpartum haemorrhage.

**Table 7:** Subgroup analyses: parity, body-mass index, baseline anaemia, and pre-delivery haemoglobin tertiles.

Analysis	N	PPH *n* (%)	aOR Duration	95% CI	*p*	Notes
Nulliparous subgroup	530	56 (10.6%)	—	—	—	AUC 0.775; cut-off 10.0 h
Multiparous subgroup	598	87 (14.5%)	—	—	—	AUC 0.772; cut-off 11.4 h
Obese (BMI ≥ 30) subgroup	268	50 (18.7%)	—	—	—	AUC 0.790; cut-off 13.5 h
Baseline anaemia (<11 g/dL)	267	29 (10.9%)	—	—	—	AUC 0.787; cut-off ~10 h
Non-anaemic (≥11 g/dL)	861	114 (13.2%)	—	—	—	AUC 0.770; cut-off ~10 h
Pre-delivery Hb tertile (lowest)	380	41 (10.8%)	1.238	1.142–1.342	<0.001	Hb 8.1–11.3 g/dL; AUC 0.758; cut-off 10.2 h
Pre-delivery Hb tertile (middle)	403	55 (13.6%)	1.195	1.120–1.275	<0.001	Hb 11.4–12.5 g/dL; AUC 0.746; cut-off 10.0 h
Pre-delivery Hb tertile (highest)	345	47 (13.6%)	1.359	1.243–1.485	<0.001	Hb 12.6–15.0 g/dL; AUC 0.818; cut-off 12.7 h

Subgroup ROC analyses present the discriminative performance of induction duration as a single continuous predictor, without adjustment. Optimal thresholds were derived using the Youden index. AUC, area under the ROC curve; BMI, body mass index; Hb, haemoglobin; PPH, postpartum haemorrhage.

## Data Availability

Restrictions apply to the availability of these data. The de-identified patient-level dataset is not publicly available because the ethics approval (Ankara Etlik City Hospital Clinical Research Ethics Committee, AEŞH-BADEK2-2026-346, 21 April 2026) and institutional data-governance policy do not authorise public release of obstetric records, and consent for open data sharing was not obtained as the study was conducted under a waiver of informed consent for retrospective electronic-record review. Aggregate study-level data and complete model coefficients sufficient to reproduce the principal analyses are provided in the manuscript, the Supplementary Materials, and the Minimal Dataset uploaded with the submission. Reasonable requests for further pseudonymised data from qualified researchers will be considered by the corresponding author, subject to a formal data-sharing agreement and additional institutional and ethics-committee approval.

## Supplementary Materials

The following supporting information can be downloaded at: https://www.mdpi.com/article/10.3390/diagnostics16121910/s1, Figure S1: STROBE flow diagram; Figure S2: ROC curves for induction duration stratified by pre-delivery haemoglobin tertiles; Supplementary Material S1: STROBE checklist.

## Abbreviations

aOR: adjusted odds ratio; ACOG: American College of Obstetricians and Gynecologists; AUC: area under the curve; BMI: body mass index (kg/m^2^); CI: confidence interval; EPV: events per variable; FIGO: International Federation of Gynecology and Obstetrics; Hb: haemoglobin; ICU: intensive care unit; IOL: induction of labour; IQR: interquartile range; NPV: negative predictive value; OR: odds ratio; PPH: postpartum haemorrhage; PPV: positive predictive value; PROM: premature rupture of membranes; QBL: quantitative blood loss; ROC: receiver operating characteristic; SD: standard deviation; STROBE: Strengthening the Reporting of Observational Studies in Epidemiology; VIF: variance inflation factor; WHO: World Health Organization.
