# Supplementary material for: Prediction of Postpartum Haemorrhage After Labour Induction: An Internally Validated 10-Hour Risk-Stratification Threshold
Source: Diagnostics (Basel). 2026 Jun 19;16(12):1910. doi: 10.3390/diagnostics16121910 (PMC13298202; doi:10.3390/diagnostics16121910)
Supplement: Supplementary file 1 [file diagnostics-16-01910-s001.zip › diagnostics-4366452-supplementary.pdf]

Supplementary Materials

Prediction of Postpartum Haemorrhage after Labour Induction: An Internally Validated 10-Hour Risk-Stratification Threshold

Supplementary Figure S1. STROBE Flow Diagram

Supplementary Figure S1. STROBE Flow Diagram

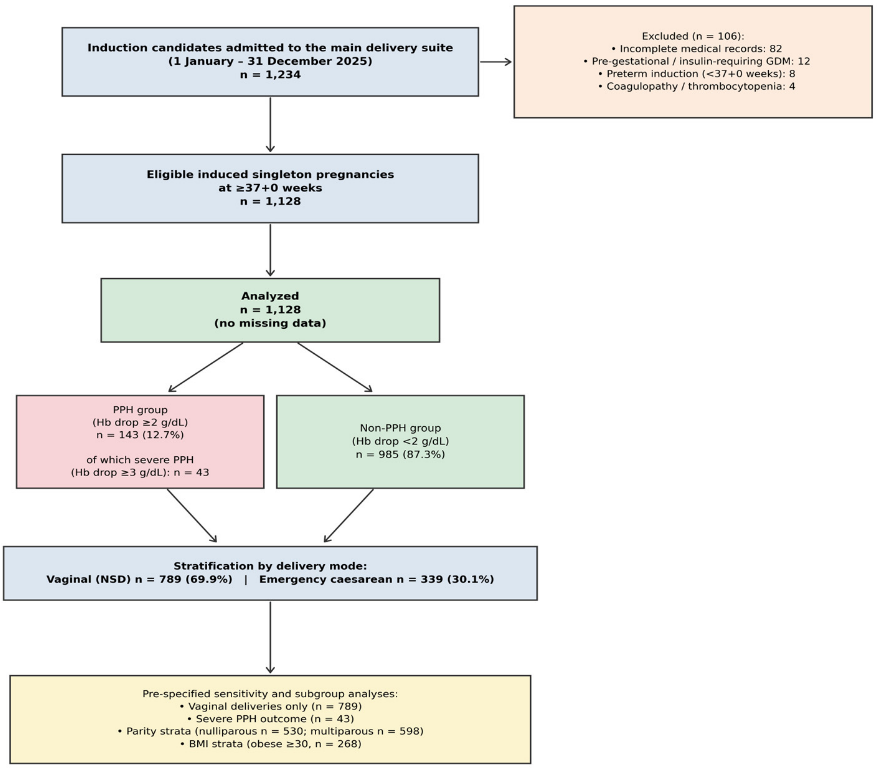

Primary outcome: Postpartum haemorrhage (PPH) = haemoglobin drop ≥2 g/dL from pre-delivery to 6–24 h postpartum.
Severe PPH = Hb drop ≥3 g/dL.
Patients managed under the institutional perinatology service (severe pre-eclampsia/HELLP, placenta praevia/accreta, multiple gestation, fetal growth restriction, major congenital anomalies) and those with previous caesarean delivery were not part of the main-delivery-suite induction population screened for this study.

Supplementary Figure S2. ROC curves for induction duration stratified by pre-delivery haemoglobin tertiles

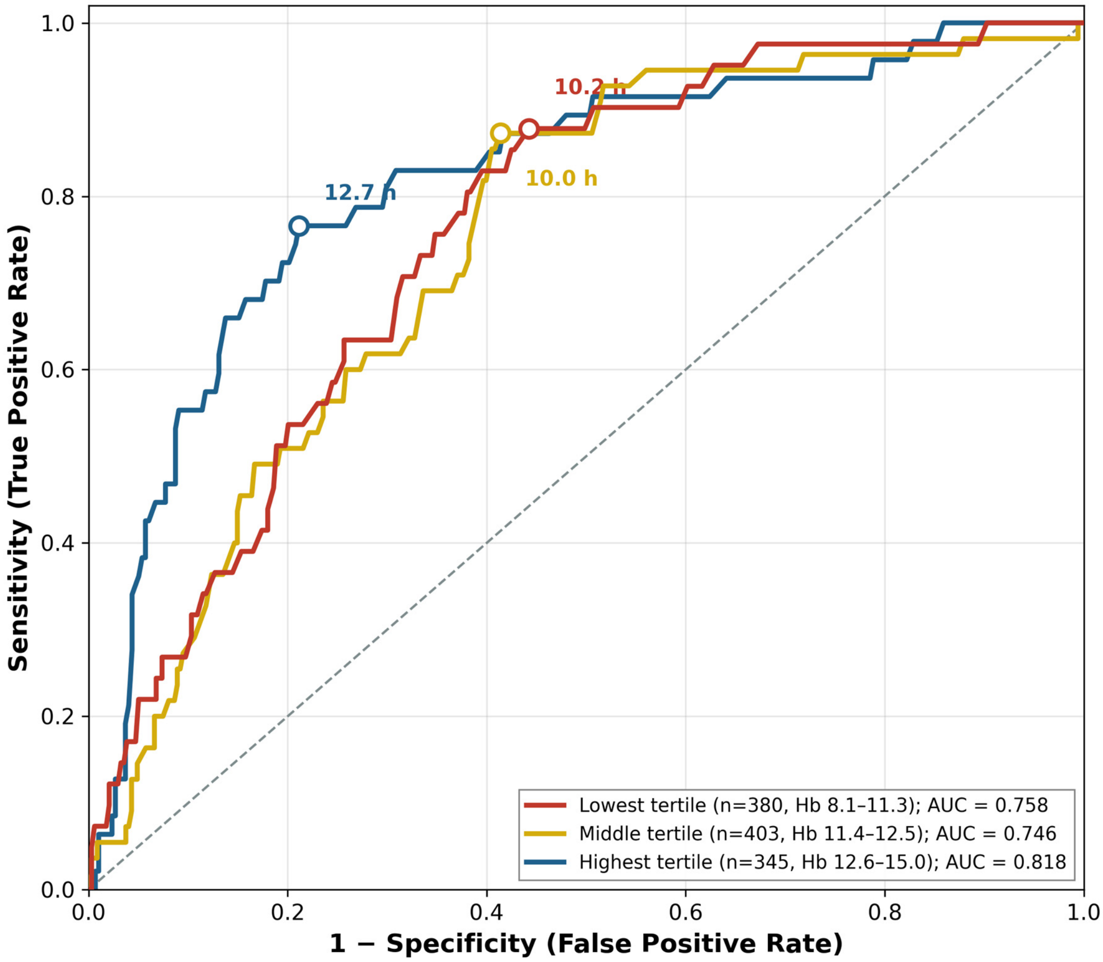

Receiver operating characteristic curves for total induction duration as a univariate predictor of postpartum haemorrhage, stratified by pre-delivery haemoglobin tertiles (lowest tertile: 8.1–11.3 g/dL, n = 380; middle tertile: 11.4–12.5 g/dL, n = 403; highest tertile: 12.6–15.0 g/dL, n = 345). Open circles mark the Youden-optimal cut-off within each tertile (10.2, 10.0, and 12.7 hours respectively). Discriminative ability was preserved across all three tertiles (AUCs 0.746–0.818), with the threshold remaining essentially identical in the lowest two tertiles and shifting upward only in the highest tertile. The likelihood-ratio test for the duration × Hb-tertile interaction (after adjustment for age, parity, and emergency caesarean) yielded χ^2 = 5.20, df = 2, p = 0.074, indicating a borderline trend rather than a definitive interaction.

Supplementary Material S1. STROBE Checklist

The following checklist documents adherence of this manuscript to the Strengthening the Reporting of Observational Studies in Epidemiology (STROBE) Statement for cohort studies (von Elm et al., 2008).

#	Item	Recommendation	Reported
1a	Title/abstract	Indicate study design in title or abstract	Title: "Retrospective Cohort Study"
1b	Title/abstract	Balanced summary in abstract	Structured abstract (Objective, Methods, Results, Conclusions)
2	Background	Explain scientific background and rationale	Introduction paragraphs 1–3
3	Objectives	State pre-specified objectives and hypotheses	Introduction paragraph 4 (3 a priori objectives)
4	Study design	Present key elements of study design	Methods: Study design and setting
5	Setting	Describe setting, locations, and relevant dates	Methods: Study design and setting (1 Jan – 31 Dec 2025)
6a	Participants	Eligibility criteria and sources of selection	Methods: Eligibility criteria
6b	Participants	Matching criteria	Not applicable
7	Variables	Define all outcomes, exposures, predictors, confounders	Methods: Outcomes and Data collection
8	Data sources/ measurement	Sources of data and methods of assessment	Methods: Data collection
9	Bias	Efforts to address potential sources of bias	Methods: dual extraction; pre-specified sensitivity analyses
10	Study size	Explain how study size was arrived at	Methods: Statistical analysis (EPV and Riley criteria)
11	Quantitative variables	Handling of quantitative variables	Methods: Statistical analysis
12a	Statistical methods	Statistical methods including confounding control	Methods: Statistical analysis
12b	Statistical methods	Subgroup and interaction analyses	Pre-specified sensitivity and subgroup analyses
12c	Statistical methods	Missing data handling	No missing data after eligibility screening
12d	Statistical methods	Loss to follow-up	Not applicable (cross-sectional outcome)
12e	Statistical methods	Sensitivity analyses	Methods: 3 pre-specified sensitivity analyses
13a	Participants	Numbers at each stage of the study	Supplementary Figure S1
13b	Participants	Reasons for non-participation	Supplementary Figure S1
13c	Participants	Flow diagram	Supplementary Figure S1
14a	Descriptive data	Characteristics of study participants	Table 1
14b	Descriptive data	Missing data per variable	No missing data
14c	Descriptive data	Follow-up time	Not applicable

15	Outcome data	Outcome events or summary measures	Results paragraph 2
16a	Main results	Unadjusted and adjusted estimates with precision	Table 3
16b	Main results	Category boundaries for continuous variables	Table 1 (BMI categories)
16c	Main results	Translation of relative to absolute risk	NPV 96.8%, PPV 22.9%
17	Other analyses	Other analyses (subgroups, sensitivity)	Table 5
18	Key results	Summarise key results with reference to objectives	Discussion paragraph 1
19	Limitations	Discuss limitations with sources of bias	Strengths and Limitations section
20	Interpretation	Cautious overall interpretation	Discussion
21	Generalisability	External validity discussion	Limitations: single-centre, single-year
22	Funding	Funding source and role	Funding statement

Reference: von Elm E, Altman DG, Egger M, et al. The Strengthening the Reporting of Observational Studies in Epidemiology (STROBE) statement: guidelines for reporting observational studies. J Clin Epidemiol. 2008;61(4):344-349. doi:10.1016/j.jclinepi.2007.11.008
